# Fatal Colonic Perforation Secondary to Non-typhoidal Salmonella Infection in an Immunocompetent Elderly Patient

**DOI:** 10.7759/cureus.109067

**Published:** 2026-05-17

**Authors:** Muhammad Zulfadhli Nor Azmi, Ummi Nadira Daut

**Affiliations:** 1 Department of Internal Medicine, Universiti Putra Malaysia, Selangor, MYS

**Keywords:** colonic perforation, elderly patient, gastroenteritis, intestinal perforation, non-typhoidal salmonella

## Abstract

Salmonella is one of the leading foodborne pathogens worldwide. Their serotypes can be classified into typhoidal, which are known to cause enteric fever, and non-typhoidal, commonly known to cause gastroenteritis or food poisoning. Salmonella gastroenteritis is typically self-limiting, and most patients recover within a few days to a week without treatment. Intestinal perforation is an exceptionally rare and fatal complication. Here, we report a case of Salmonella gastroenteritis complicated by colonic perforation. Postoperatively, the patient succumbed due to multiorgan failure and intra-abdominal sepsis. This case highlights that even non-typhoidal Salmonella infection in an immunocompetent host may lead to catastrophic complications. Early recognition of clinical deterioration and timely surgical intervention are crucial to improve outcomes.

## Introduction

Salmonellae spp. are Gram-negative, motile, non-sporulating, straight rod bacteria belonging to the family Enterobacteriaceae. They are highly pathogenic and encompass more than 2,600 serovars [1]. They remain a major cause of foodborne illness worldwide, with transmission typically occurring through the ingestion of contaminated food and water [2,3].

Non-typhoidal Salmonella infections commonly present with acute-onset diarrhea, abdominal cramps, and fever and are usually self-limiting. In contrast, typhoidal Salmonella serovars cause enteric fever, characterized by sustained high fever (39°-40°C), headache, diarrhea or constipation, loss of appetite, and relative bradycardia [4,5].

Although intestinal perforation is a well-recognized complication of typhoidal infection, colonic perforation secondary to non-typhoidal Salmonella in an immunocompetent patient is exceedingly rare [2,3,6]. A case of colonic perforation due to Salmonella Enteritidis infection was previously reported in Malaysia [7]. This case highlights the potential for severe and atypical complications arising from an otherwise self-limiting illness, underscoring the importance of early recognition and prompt intervention.

## Case presentation

A 73-year-old lady with chronic cauda equina syndrome secondary to L3/L4 spondylolisthesis, characterized by urinary incontinence with intact bowel function, presented to the emergency department with a five-day history of diarrhea associated with abdominal discomfort, lethargy, and poor oral intake. The symptoms developed one day after a family meal, during which most other attendees experienced similar gastrointestinal complaints. She also reported a fall in the toilet on the day of presentation due to worsening bilateral lower limb weakness, which had limited her ambulation since the onset of diarrhea. There was no fever, neck stiffness, blurred vision, or any seizure activity.

On arrival, she was alert and conscious with normal vital signs. Her abdomen was soft and non-tender, and her respiratory and cardiovascular examinations were unremarkable. Neurological examination revealed bilateral lower limb weakness with reduced reflexes and sensation, with intact anal tone, consistent with her underlying condition. Initial full blood count demonstrated leukocytosis with mild anemia and a normal platelet count. She also had evidence of acute kidney injury, while venous blood gas analysis showed metabolic acidosis with elevated lactate (Table 1). HIV screening was negative. She was admitted with a diagnosis of acute infectious gastroenteritis.

**Table 1:** The patient's initial laboratory values on day one and lactate level on day seven.

Variables	Laboratory values (day 1)	Laboratory values (day 7)	Reference ranges
White blood cell count (×10⁹/L)	16	N/A	4-10
Hemoglobin (g/dL)	11.3	N/A	12-15
Platelet (×10⁹/L)	278	N/A	150-410
Urea (mmol/L)	15	N/A	2.76-8.07
Creatinine (µmol/L)	105	N/A	44-80
Lactate (mmol/L)	2.9	3.49	0.36-1.39

The patient received intravenous ceftriaxone and metronidazole for four days before escalation to intravenous piperacillin/tazobactam due to persistent diarrhea. Stool culture grew Salmonella spp., which was sensitive to ampicillin, ciprofloxacin, and co-trimoxazole, while blood cultures showed no growth. Orthopedics was consulted regarding her cauda equina syndrome; however, she declined surgical intervention.

**Figure 1:**
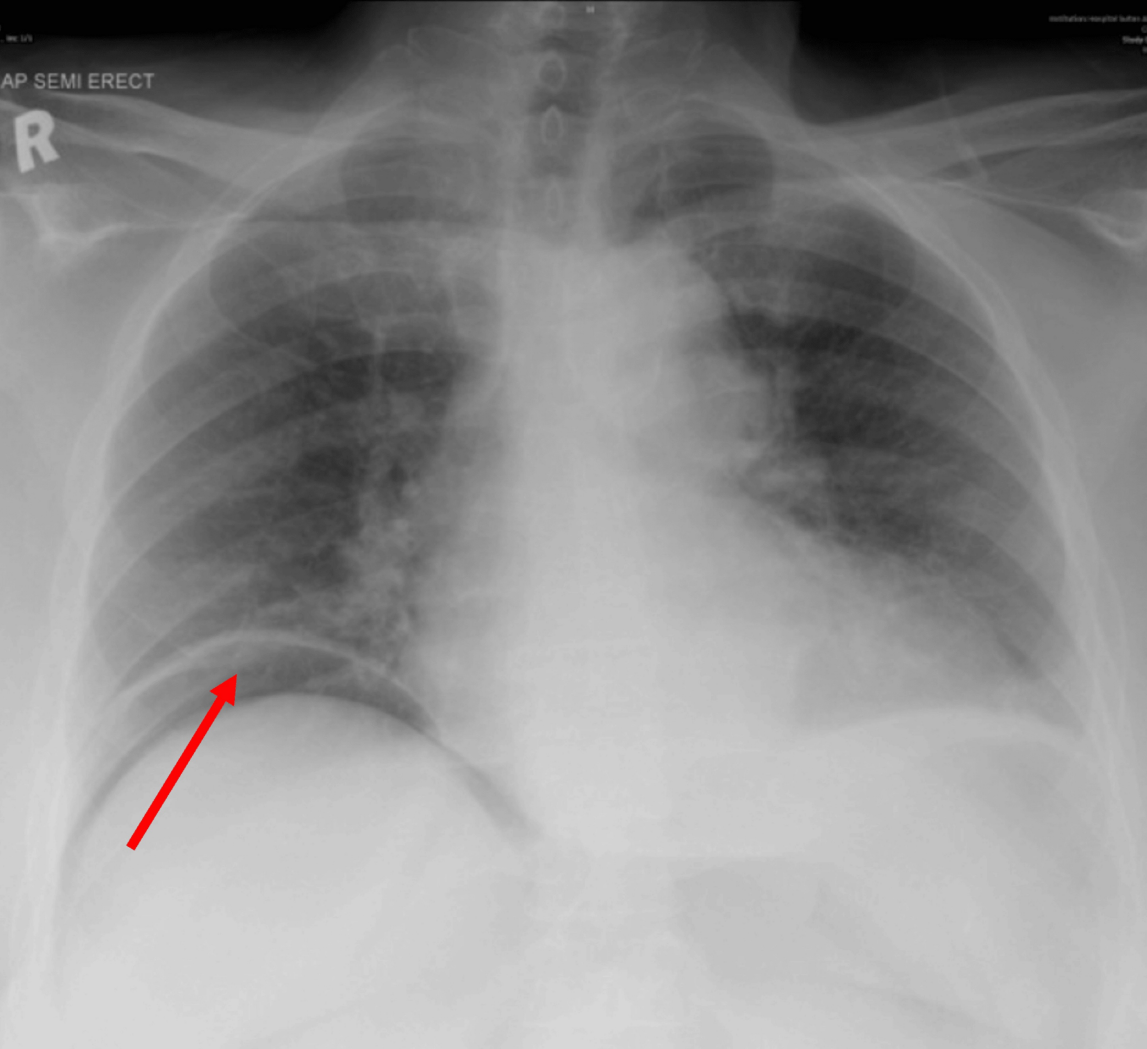
Chest radiograph demonstrating air under the right hemidiaphragm (arrow), consistent with pneumoperitoneum.

**Figure 2:**
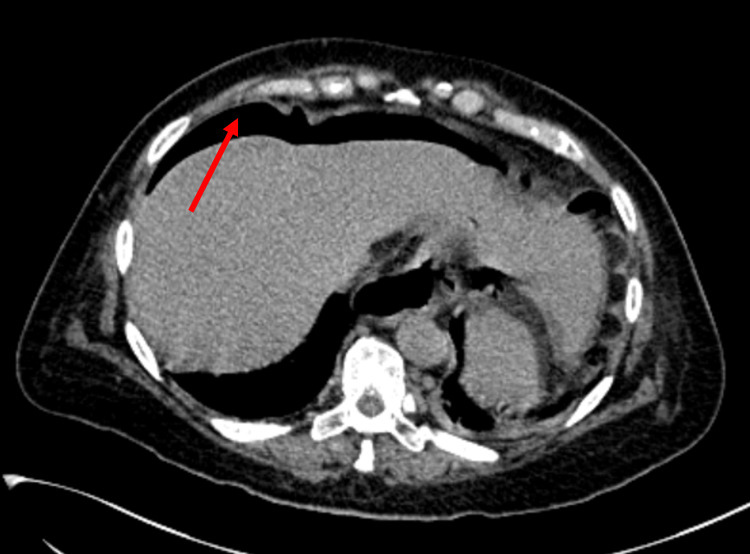
Computed tomography of the abdomen demonstrating free intraperitoneal air (arrow), consistent with pneumoperitoneum.

On day seven of admission, she developed severe abdominal pain, hypotension, and rising lactate levels. Her abdomen became tense and distended. Chest radiograph showed air under the diaphragm, while abdominal radiograph demonstrated dilated large bowel loops (Figure 1). Urgent computed tomography (CT) of the abdomen revealed a perforated viscus (Figure 2). She further deteriorated later that evening and was intubated for airway protection.

She underwent an emergency exploratory laparotomy, which revealed multiple perforation sites in the sigmoid colon, with polyposis coli noted in the mucosa. There was gross fecal contamination in all quadrants, predominantly in the pelvis, with interloop small bowel abscesses. The transverse colon appeared dilated and slightly dusky; therefore, transection and colostomy were performed. Histopathological examination (HPE) of the colon demonstrated a perforated colon with background acute fulminant colitis and no evidence of malignancy.

Postoperatively, she deteriorated further, requiring continuous venovenous hemodialysis and a second exploratory laparotomy due to intraperitoneal bleeding with a non-viable colostomy. Repeat blood cultures remained negative, while peritoneal fluid culture grew extended-spectrum beta-lactamase (ESBL) *Klebsiella pneumoniae*,* *which was sensitive to meropenem, ertapenem, and imipenem. Antibiotic therapy was escalated to intravenous meropenem. Despite aggressive management, she succumbed after 11 days in the intensive care unit due to multiorgan failure secondary to intraperitoneal sepsis.

## Discussion

Salmonella is a major foodborne pathogen with a significant impact on global health and economic burden. The World Health Organization (WHO) describes Salmonella as one of the four most important causes of diarrheal disease. Annually, an estimated 200 million to 1 billion cases of Salmonella infection occur worldwide, including approximately 93 million cases of gastroenteritis and 155,000 deaths. Approximately 85% of cases are associated with the consumption of contaminated food. Typhoidal Salmonella causes typhoid fever, whereas non-typhoidal Salmonella (NTS) typically causes gastroenteritis. While Salmonella gastroenteritis is usually self-limiting in immunocompetent individuals, it can become invasive in patients with HIV, malaria, malnutrition, sickle cell anemia, or in the elderly.

Intestinal perforation is a rare but serious complication of Salmonella infection, particularly in children and in patients from endemic regions [8]. It is most commonly associated with typhoidal Salmonella; however, several cases of perforation due to non-typhoidal Salmonella have been reported [2,3,6]. For instance, Hew et al. described a case of non-typhoidal Salmonella Typhimurium causing intestinal perforation in a 40-year-old man with no predisposing risk factors [6].

The pathophysiology of intestinal perforation in Salmonella infection is multifactorial. In typhoidal disease, bacterial invasion of Peyer’s patches leads to necrosis and ulceration, ultimately resulting in perforation [9]. In contrast, NTS-related perforation is thought to involve a combination of severe inflammatory response, cytokine-mediated tissue injury, and microvascular compromise leading to bowel ischemia [10]. Persistent diarrhea and dehydration may further exacerbate hypoperfusion, increasing the risk of ischemic necrosis and subsequent perforation.

In this patient, several factors may have contributed to bowel perforation. Advanced age is a well-recognized risk factor for invasive Salmonella infection due to immunosenescence and impaired host defense mechanisms [4,5]. In addition, intraoperative findings of polyposis coli suggest underlying structural bowel pathology, which may have predisposed to weakened mucosal integrity and increased susceptibility to transmural injury.

The involvement of the sigmoid colon in this case may be explained by its relatively vulnerable blood supply, particularly at watershed areas, making it more susceptible to ischemic injury during systemic illness and hypoperfusion states [11]. This is especially relevant in elderly patients with compromised vascular reserve.

Furthermore, the development of secondary infection with extended-spectrum beta-lactamase (ESBL)-producing *Klebsiella pneumoniae *significantly worsened the clinical course. Secondary intra-abdominal sepsis following perforation is associated with high morbidity and mortality, particularly when caused by multidrug-resistant organisms, despite appropriate escalation of antimicrobial therapy [12].

This case highlights several important clinical lessons. Worsening abdominal pain, abdominal distension, rising lactate levels, and hemodynamic instability in a patient with presumed gastroenteritis should prompt urgent evaluation for complications such as bowel ischemia or perforation. Early imaging, timely surgical intervention, and aggressive sepsis management are critical to improving outcomes.

## Conclusions

Although non-typhoidal Salmonella infections are typically self-limiting, this case demonstrates that they may rarely progress to life-threatening complications such as colonic perforation, particularly in vulnerable populations such as the elderly. Age-related immunosenescence, together with underlying colonic pathology as suggested by polyposis coli in this patient, may have contributed to increased susceptibility to invasive disease and bowel wall compromise. This report adds to the limited literature on non-typhoidal Salmonella-associated colonic perforation in immunocompetent individuals and highlights the need for heightened clinical awareness of this rare but catastrophic complication.

This case underscores the importance of maintaining a high index of suspicion in patients with persistent diarrhea who develop worsening abdominal pain, abdominal distension, rising lactate levels, or hemodynamic instability. Early imaging, prompt surgical intervention, and aggressive multidisciplinary management are essential to improving outcomes. Clinicians should remain vigilant, as even seemingly benign gastrointestinal infections can deteriorate rapidly, particularly in high-risk patients, and timely recognition of red-flag signs is crucial for preventing fatal outcomes.
